# Gender-Specific Efficacy Revealed by Head-to-Head Comparison of Pasireotide and Octreotide in a Representative In Vivo Model of Nonfunctioning Pituitary Tumors

**DOI:** 10.3390/cancers13123097

**Published:** 2021-06-21

**Authors:** Sebastian Gulde, Tobias Wiedemann, Mathias Schillmaier, Isabel Valença, Amelie Lupp, Katja Steiger, Hsi-Yu Yen, Stephen Bäuerle, Johannes Notni, Raul Luque, Herbert Schmid, Stefan Schulz, Donna P. Ankerst, Franz Schilling, Natalia S. Pellegata

**Affiliations:** 1 Institute for Diabetes and Cancer, Helmholtz Zentrum München, 85764 Neuherberg, Germany; sebastian.gulde@helmholtz-muenchen.de (S.G.); tobias.wiedemann@helmholtz-muenchen.de (T.W.); isabel.valenca@helmholtz-muenchen.de (I.V.); 2 Joint Heidelberg-IDC Translational Diabetes Program, Heidelberg University Hospital, 69120 Heidelberg, Germany; 3 Department of Nuclear Medicine, School of Medicine, Technical University of Munich, 80333 Munich, Germany; mathias.schillmaier@tum.de (M.S.); franz.schilling@tum.de (F.S.); 4 Institute of Pharmacology and Toxicology, Jena University Hospital, Friedrich Schiller University Jena, 07743 Jena, Germany; Amelie.Lupp@med.uni-jena.de (A.L.); Stefan.Schulz@med.uni-jena.de (S.S.); 5 Institute of Pathology, School of Medicine, Technical University of Munich, 80333 Munich, Germany; katja.steiger@tum.de (K.S.); hsiyuyen@gmail.com (H.-Y.Y.); johannes.notni@tum.de (J.N.); 6 Department of Mathematics, Technical University of Munich, 85748 Garching, Germany; stephen.baeuerle@googlemail.com (S.B.); ankerst@tum.de (D.P.A.); 7 Experimental Radiopharmacy, Clinic for Nuclear Medicine, University Hospital Essen, 45147 Essen, Germany; 8 Department of Cell Biology, Physiology, and Immunology, Maimonides Institute for Biomedical Research of Córdoba (IMIBIC), University of Córdoba and Hospital Universitario Reina Sofía (HURS), 14004 Cordoba, Spain; bc2luhur@uco.es; 9 Centro de Investigación Biomédica en Red de Fisiopatología de la Obesidad y Nutrición (CIBERobn), 14004 Cordoba, Spain; 10 Department of Oncology Research, Novartis Institute for BioMedical Research, Novartis Pharma AG, 4033 Basel, Switzerland; herbert.schmid@novartis.com; 11 Department of Biology and Biotechnology “L. Spallanzani”, University of Pavia, 27100 Pavia, Italy

**Keywords:** nonfunctioning pituitary tumors, octreotide, pasireotide, somatostatin receptors, MRI, sex differences in drug response

## Abstract

**Simple Summary:**

No effective medical therapy exists for residual/recurrent nonfunctioning pituitary tumors (NFPTs). First-generation somatostatin analogs (SSAs) like octreotide targeting somatostatin receptor type 2 (SSTR2) are the mainstay therapy for functioning PTs, but have shown little effect in NFPTs. This is in agreement with an SSTR profile characterized by low SSTR2, and high SSTR3 levels in the latter. Pasireotide a multi-SSTR-preferring SSA, should be effective against NFPTs. To test this hypothesis, we conducted a head-to-head comparison of octreotide and pasireotide in the only spontaneous in vivo model of NFPTs (MENX rats), which recapitulates the human disease. Pasireotide showed a superior anti-tumor effect vs. octreotide, especially in females. Interestingly, *Sstr3* levels were higher in female vs. male NFPTs. A sex-related SSTR3 expression may extend to human NFPTs, thereby representing a tool for patient stratification. Our results have translational relevance for the medical treatment of patients with residual/recurrent NFPTs currently lacking efficacious therapeutic options.

**Abstract:**

Invasive nonfunctioning pituitary tumors (NFPTs) are non-resectable neoplasms associated with frequent relapse and significant comorbidities. Current treatments, including somatostatin receptor 2 (SSTR2)-directed somatostatin analogs (SSAs), often fail against NFPTs. Thus, identifying effective therapies is clinically relevant. As NFPTs express SSTR3 at high levels, pasireotide, a multireceptor-targeted SSA, might be beneficial. Here we evaluated pasireotide in the only representative model of spontaneous NFPTs (MENX rats) in vivo. Octreotide long-acting release (LAR), pasireotide LAR, or placebo, were administered to age-matched, tumor-bearing MENX rats of both sexes for 28 d or 56 d. Longitudinal high-resolution magnetic resonance imaging monitored tumor growth. While tumors in placebo-treated rats increased in volume over time, PTs in drug-treated rats displayed significant growth suppression, and occasional tumor shrinkage. Pasireotide elicited stronger growth inhibition. Radiological responses correlated with tumors’ proliferation rates. Both SSAs, but especially pasireotide, were more effective in female vs. male rats. Basal *Sstr3* expression was significantly higher in the former group. It is noteworthy that female human NFPTs patients also have a trend towards higher SSTR3 expression. Altogether, our studies provide the rationale for testing pasireotide in patients with residual/recurrent NFPTs. If confirmed, the sex-related SSTR3 expression might be used as criteria to stratify NFPTs patients for treatment with pasireotide.

## 1. Introduction

Pituitary adenomas are the third most common intracranial tumors and are characterized by variable clinical behavior [1]. Pituitary tumors (PTs) pose a considerable impact on patient functional status and mortality [2,3]. One-third of all PTs do not hypersecrete any pituitary hormone and are thus called non-functioning (NFPTs). In over 80% of cases, they derive from gonadotroph cells [4]. Transsphenoidal surgery is the mainstay treatment for patients with PTs. Due to the lack of symptoms secondary to hormone hypersecretion, patients with NFPTs come to medical attention when they show symptoms of “mass effects” as, e.g., headaches caused by tumors compressing surrounding structures [5,6]. At this stage, more than 40% of NFPTs invade parasellar structures (sphenoidal/cavernous sinuses) complicating complete surgical removal. This leads to relapse in up to 30% of cases at 10 years after the first surgery [7,8]. Radiotherapy is the only treatment for tumor remnants, but it associates with long-term side effects [9,10]. Medical treatment with dopamine agonists, first-generation somatostatin analogues (SSAs) and temozolomide have produced disappointing results for the treatment of NFPTs [6,11]. Altogether, effective medical therapies are needed for the post-operative management of residual NFPTs given their high relapse rate.

Neuroendocrine tumors (NETs), including PTs, express somatostatin receptors (SSTR1–5), which are G-protein coupled receptors bound and activated by the hormone somatostatin (SST) [12,13]. SSTR receptors have been exploited as therapeutic targets for NETs given that their activation suppresses hormone secretion in several SST target tissues, as well as inhibits the proliferation of normal and tumor cells [13]. Indeed, treatment with clinically available SSAs normalizes hormone secretion, shrinks tumors, and improves clinical symptoms in various NETs [8,13]. The currently available SSAs have different binding affinities for the various SSTRs: octreotide and lanreotide (first-generation SSAs) have a higher affinity for SSTR2, whereas the multi-receptor ligand pasireotide has an affinity for SSTR5 > SSTR2 > SSTR3 [14]. We and others have shown that NFPTs express SSTR3 at higher levels than SSTR2 [15,16,17,18], and this might explain why they do not respond to first-generation, SSTR2-directed SSAs. Instead, NFPTs are expected to benefit from the treatment with the multi-receptor SSA pasireotide.

To test the hypothesis that a multi-receptor SSA is more effective against NFPTs than an SSTR2-directed agent, we conducted a head-to-head comparison of octreotide and pasireotide in the only spontaneous, endogenous model of NFPTs currently available (=MENX rats). Indeed, rats affected by the MENX syndrome develop with 100% penetrance gonadotroph tumors that closely resemble human NFPTs at histological, immunohistochemical, molecular and transcriptomic levels [19,20], as well as for SSTR expression. Tumor growth was monitored longitudinally by high-resolution magnetic resonance imaging (MRI). Ex vivo molecular readouts (*Sstr* expression, proliferation, apoptosis) assessed drug effects. In our endogenous NFPT model, pasireotide was more potent at suppressing tumor growth than octreotide, thereby setting the rationale for testing pasireotide against the cognate human tumors. Interestingly, we observed a clear sex-related difference in drug response, partially explained by a sex-dependent expression of *Sstr3*, which might also be relevant for human NFPT patients stratification.

## 2. Results

### 2.1. Efficacy of Octreotide LAR vs. Pasireotide LAR against Endogenous NFPTs In Vivo

**Figure 1:**
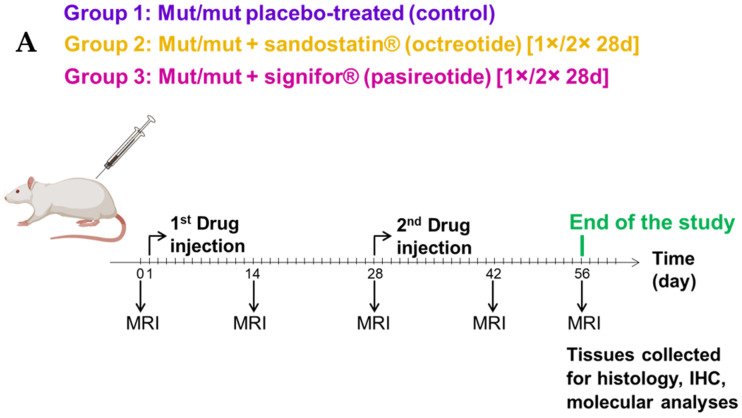

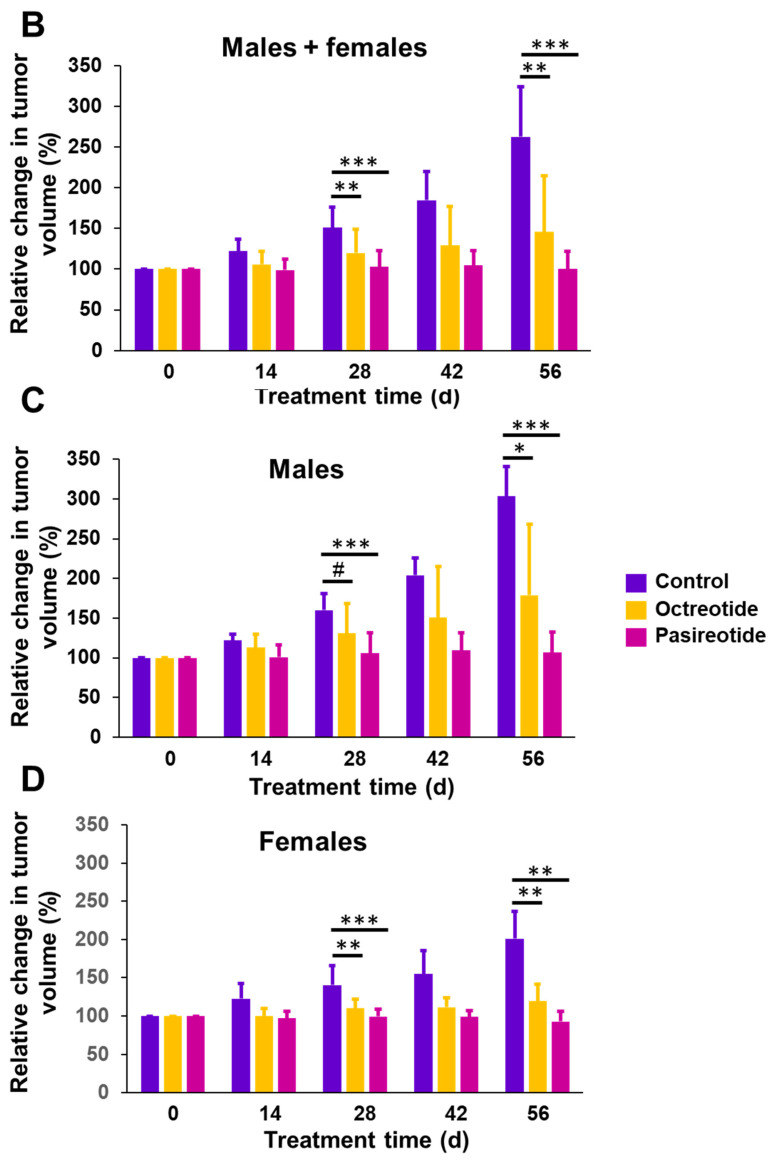
Head-to-head in vivo evaluation of octreotide LAR (sandostatin^®^) and pasireotide LAR (signifor^®^). (**A**) Treatment scheme. MENX-affected rats at the age of 5.5 months were injected with sandostatin^®^ and signifor^®^ 1× or 2× every 28 days at the indicated dose. MRI was performed every 14 days. At the end of treatment, animals were sacrificed, and tissues were collected for histological and molecular analyses. (**B**–**D**) Relative changes in tumor volume as measured by MRI. Rats of the 3 treatment groups were scanned every 14 days and the tumor volume was normalized against the volume at day 0. In (**B**), male and female rat tumors are combined. In (**C**) only males and in (**D**) only female rat tumors are shown. Data presented are the mean ± SEM. #, not significant; *, *p* < 0.05; **, *p* < 0.001; ***, *p* < 0.0001.

We treated age-matched MENX rats of both sexes with octreotide long-acting release (LAR) (sandostatin^®^, Novartis Pharma GmbH, Nürnberg, Germany), pasireotide LAR (signifor^®^, Novartis Pharma GmbH, Nürnberg, Germany) or placebo (control group). Rats were treated for 28 days (d) (control group *n* = 9; octreotide *n* = 8; pasireotide *n* = 6) or for 56 d (control group *n* = 10; octreotide *n* = 9; pasireotide *n* = 17) following the scheme illustrated in Figure 1A (Table S1 lists the rats used for the study). Tumor volume during treatment was assessed by magnetic resonance imaging (MRI) and normalized to the volume at day 0 for each animal (Figure 1B; Tables S2–S4). Given that sex-related differences in the response to common anti-cancer drugs have been reported [21], we also analyzed males and females separately (Figure 1C,D). Longitudinal MRI scans of tumors representative of each treatment group (56 d treatment) are shown in Figure S1.

**Figure 2:**
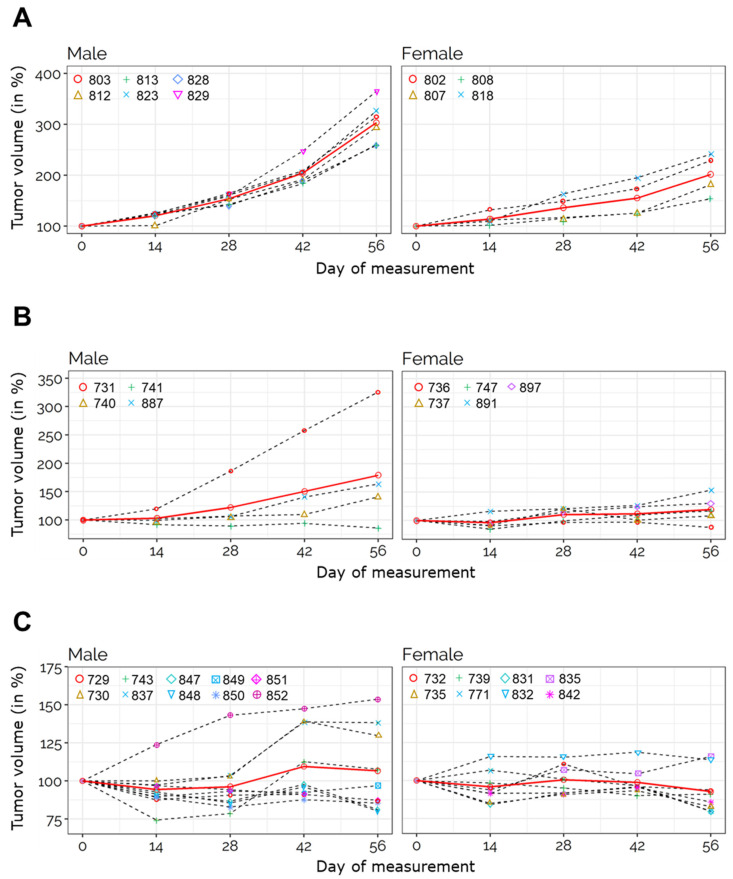
Trace plots of relative tumor volume in the various groups at 56 d post-treatment. Trace plots of tumor volume changes for mutant rats of the different treatment groups relative to the volume at day 0 for each animal. Left, male rats. Right, female rats. (**A**) Trace plot for placebo-treated mutant rats. For statistical analysis an LME model with mean structure of log(rel_volume) ~ 1 + day + male + male × day + day^2^ + male × day^2^ achieved the best fit (in red). Difference in time slopes of male and female rats is significant (*p* = 0.0420 when compared to associated F-distribution). (**B**) Trace plots of relative tumor for octreotide-treated mutant rats. For statistical analysis an LME model with mean structure of log(rel_volume) ~ 1 + day achieved the best fit (in red). Time effect is significant (*p* = 0.0017 when compared to associated F-distribution), gender effects are not detected as being significant. (**C**) Trace plots of relative tumor for pasireotide-treated mutant rats. For statistical analysis, the LME model with mean structure of log (rel_volume) ~ 1 achieved the best fit (in red). All other predictors were not significant.

Every tumor of placebo-treated (control) rats increased in volume over the course of the experiment, with tumors reaching an average of +300% increase in size vs. day 0 in male rats, and a 2-fold increase (+200%) in the females (Figure 1B–D). Tumor volume increased quadratically in the 56-day placebo-treated group, with faster increases among males compared to females (*p*-value = 0.04) (Figure 2A, Figure S2).

In contrast to the placebo group, animals treated with the tested drugs experienced suppression of tumor growth, and in some cases reduction in tumor volume. Specifically, octreotide suppressed tumor growth in rats of both sexes up to 56 d post-treatment (Figure 1B) and was particularly effective at inhibiting tumor growth in female rats. After 28 d of treatment with octreotide female mutant rats displayed on average a tumor volume increase of +9%, and an increase of +19% after 56 d (*p* = 0.00403 vs. controls at both 28 d and 56 d) (Figure 1B,D and Figure 2B, Figure S2). PTs in males increased their relative volume up to +40% on average at the 28 d time point, and up to +79% at the 56 d time point (*p* = 0.024 vs. controls at both 28 d and 56 d) (Figure 1B,C and Figure 2B, Figure S2). Upon octreotide treatment, 4 out of 17 (23.5%) NFPTs showed a reduction in tumor volume (considering both treatment durations).

**Figure 3:**
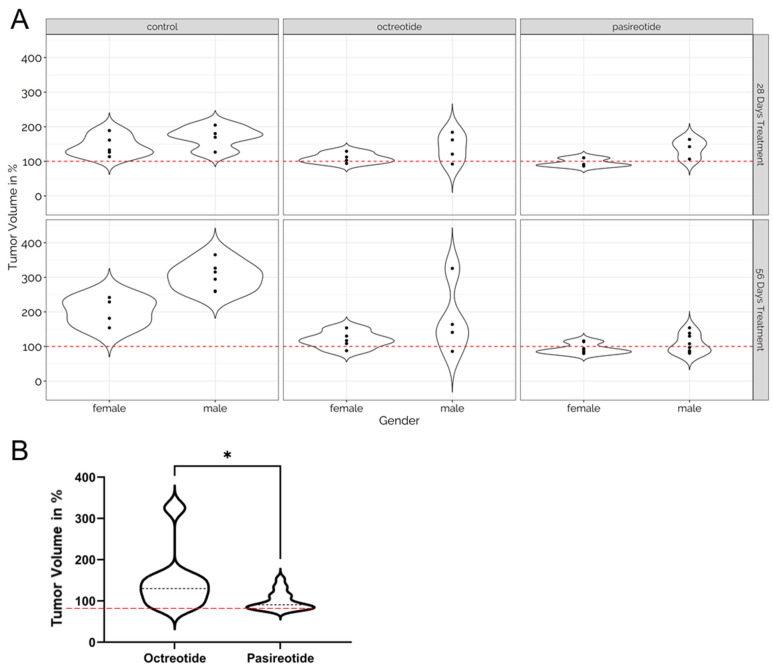
Distribution of the relative tumor volume at the end of the experiment (28 d/56 d). (**A**) Distribution of relative tumor volume changes in rats treated for 28 d (upper row) and 56 d (bottom row) vs. day 0, grouped by treatment and gender. The red dashed line indicates 100%. The distribution is shown as a violin plot. (**B**) Distribution of relative tumor volume changes in octreotide and pasireotide treated rats (56 d) vs. day 0. The red dashed line indicates 100%. The distribution is shown as a violin plot, the black dashed line indicates the median. For statistical analysis, the LME model was used. *, *p* = 0.0088.

Treatment of MENX rats with pasireotide elicited a more pronounced response in both sexes combined when compared to octreotide (*p* < 0.001 vs. controls at both 28 d and 56 d) (Figure 1B and Figure 2C, Figure S2). Pasireotide treatment for 56 d led to a slight reduction of relative tumor volume in females (−7.2% on average), and a slight increase in males (+7% on average). Figure 3A shows the relative tumor volume of each rat at the end of the experiment. Animals were grouped by sex and treatment. Average tumor growth in pasireotide-treated rats was significantly lower than in animals administered with octreotide (*p* = 0.0088, Figure 3B). Comparisons of longitudinal percent change in tumor volume according to study time, to treatment/control group and sex indicated pasireotide to have the greatest impact on tumor suppression (Figure S3), which was stronger in females compared to males (Figure S4). Pasireotide administration decreased tumor volume in 12 out of 24 (50%) tumors (considering both treatment durations).

Altogether, in our model, pasireotide showed a superior tumor growth control than octreotide (Figure 2 and Figure 3).

### 2.2. Proliferation Rates of NFPTs in the Three Treatment Groups

**Figure 4:**
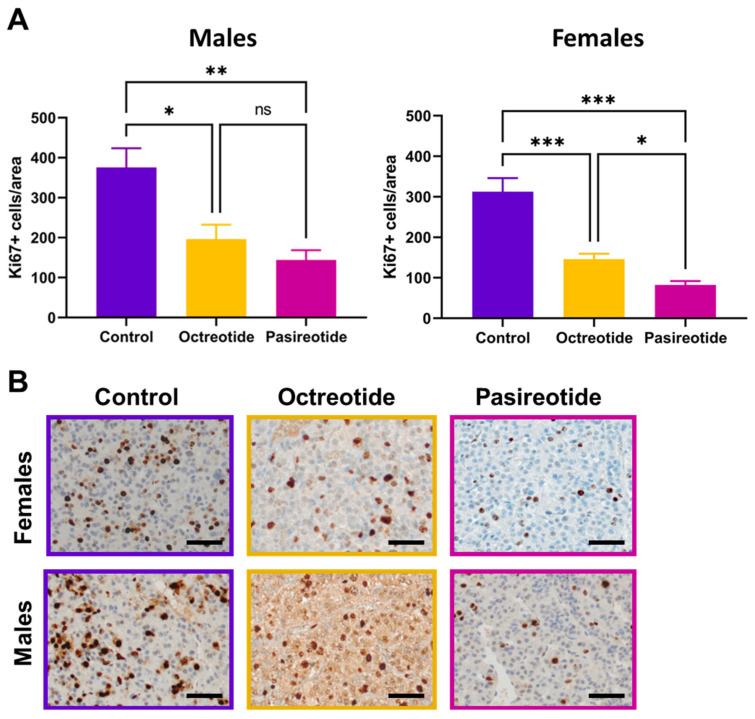
Proliferation of PTs following treatment. (**A**) Number of Ki67-positive cells per 100,000 m^2^ in PTs of rats belonging to the three treatment groups and two sexes after 56 d of treatment. Shown is the mean ± SEM. n.s., not significant; *, *p* < 0.05; **, *p* < 0.001; ***, *p* < 0.0001. (by one-way ANOVA) (**B**) Representative immunohistochemical stainings conducted with the anti-Ki67 antibody. Original magnification: ×400. Size bar: 50 μm.

At the end of treatment, pituitary tissues were collected for ex vivo analyses. Staining for Ki67 was performed on all tumors of rats treated for 56 d with either placebo or SSAs. Subsequently, the number of Ki67-positive cells per 100,000 µm^2^ was counted. Placebo-treated (control) PTs showed an average of 312 ± 33.5 (females) and 376 ± 48 (males) Ki67-positive cells per area (Figure 4). Not surprisingly, in the control group, there was a positive trend between the number of Ki67-positive cells and absolute tumor volume in males and females (*p* = 0.01727). In tumors of rats treated with octreotide, the numbers of Ki-67-positive cells dropped to an average of 146 ± 13.5 (*p* < 0.0001 vs. controls) for females and 197 ± 35.5 (*p* = 0.017 vs. controls) for males. The decrease in Ki67-positive cells per area was even more pronounced after pasireotide administration, reaching on average 87 ± 11 (*p* < 0.0001 vs. controls) for female rats and 144 ± 24 (*p* = 0.0003 vs. controls) for the males (Figure 4; Figure S5). The difference in the number of Ki67-positive cells between placebo- and drug-treated groups was significant (*p* = 7.76 × 10^−9^ for octreotide and pasireotide combined), as well as for the drugs alone (*p* = 8.8 × 10^−6^ for octreotide and *p* = 2.78 × 10^−11^ for pasireotide) (Figure 4; Figure S5). These data are in agreement with the tumor growth behavior as determined by MRI. Indeed, PTs proliferated less in females than in males (in the control group) (Figure 4). Moreover, the most effective SSA at suppressing tumor growth during treatment was pasireotide, and this correlated with the strongest reduction in PT cell proliferation (Figure 4). Octreotide was less potent at inhibiting tumor volume growth, and also less efficient at inhibiting cell proliferation.

### 2.3. Expression of Sstrs and Apoptosis Markers in Rat NFPTs

Expression levels of the *Sstr* genes were estimated in rat PTs at the end of treatment (56 d) by assessing copy numbers for each transcript (absolute quantification).

**Figure 5:**
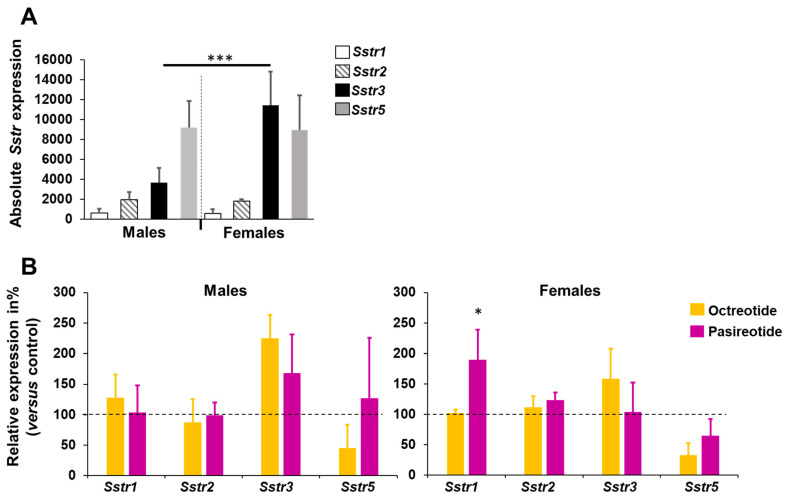
SSTR gene expression in rat PTs. (**A**) Absolute quantification of mRNA copy number/cell for *Sstr1*, *2*, *3*, *5* genes in placebo-treated control rats of both sexes. (**B**) Relative expression of the *Sstr1*, *2*, *3*, *5* genes in each treatment group (octreotide and pasireotide) compared to the control group, arbitrarily set to 100%. (**A**,**B**) Shown is the average ± SEM. *, *p* = 0.0059; ***, *p* < 0.0001.

In the control group, the most highly expressed gene in both sexes was *Sstr5* (Figure 5A). When considering the sexes separately, we noticed that females have a statistically significantly higher amount of *Sstr3* mRNA (3-fold) when compared with male rats (*p* = 0.00132) (Figure 5A). Interestingly, there was a negative linear correlation between relative tumor volume and *Sstr3* expression (*p* = 0.019), suggesting that PTs with higher *Sstr3* levels grew less rapidly (Figure S6).

Upon SSA administration, the levels of some *Sstr* genes changed when compared to the placebo-treated controls. While octreotide treatment led to a decrease in the expression of *Sstr5* in both sexes, pasireotide led to higher levels of *Sstr1* and *Sstr2* mRNAs in females and to higher *Sstr3* levels in males (Figure 5B). These trends were however not significant, expect the increase of *Sstr1* in females treated with pasireotide.

**Figure 6:**
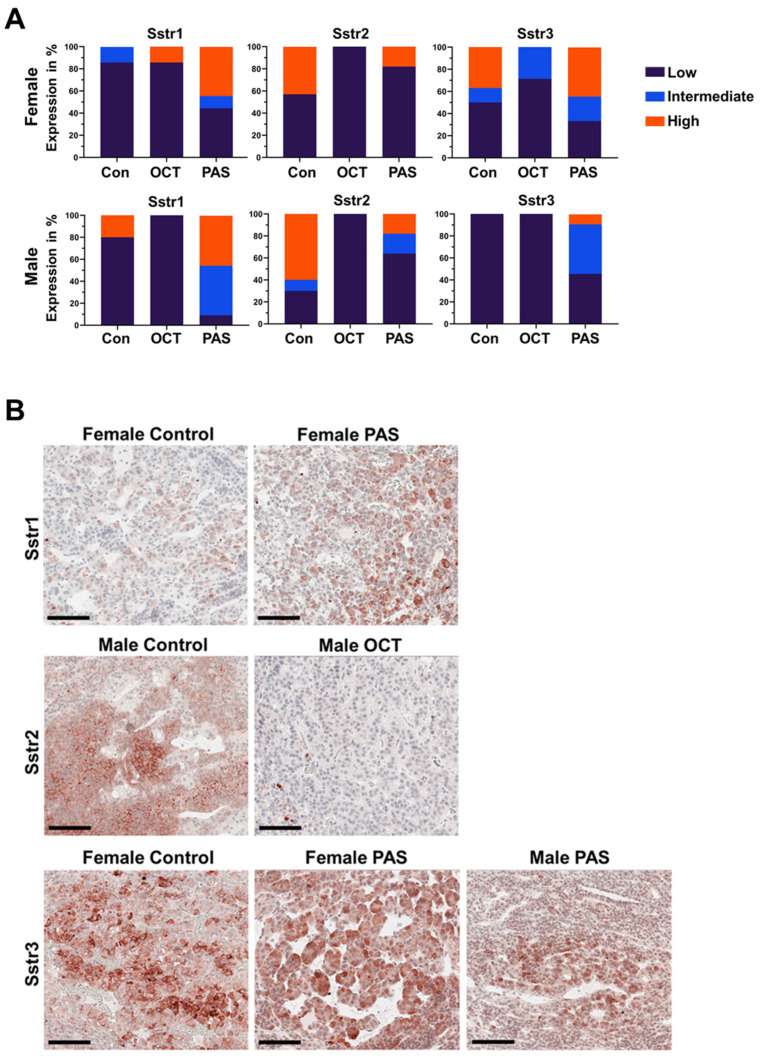
Expression of Sstr receptors in rat PTs following treatment. (**A**) Scoring of the expression of Sstr1,2 and 3 in rat tumors following treatment. In each treatment group/gender at least three tumors were stained. (**B**) Shown are representative samples from the treatment groups that showed changes in Sstr protein expression in their tumors upon treatment. PAS, pasireotide; OCT, octreotide. Original magnification: ×200. Size bar: 200 μm.

A subset of the PTs from the three animal groups was also stained with antibodies against Sstr1, 2, and 3 using immunohistochemistry (IHC). The anti-SSTR5 antibody gave unreliable results on rat tissues and is therefore not included. Octreotide mainly affected the expression of Sstr2, which was decreased upon drug administration (Figure 6). This is in agreement with data on PT patients [22,23,24], and implies that there is no correlation between *Sstr2* mRNA and protein levels. Pasireotide treatment increased the expression of Sstr1 (in females) and Sstr3 (in males) (Figure 6), and this correlates with changes seen at the mRNA levels.

**Figure 7:**
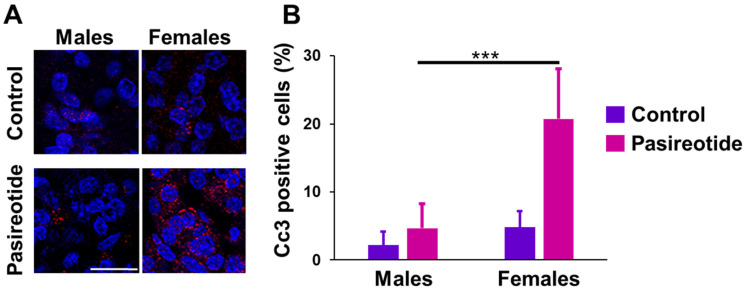
Cleaved caspase 3 (Cc3) expression in rat PTs. (**A**) Immunofluorescent staining of PT tissue sections of rats from the two groups (pasireotide-treated and control) were stained with an antibody against Cc3 (red). Nuclei were counterstained with Hoechst 33258 (blue). Shown are sections representative of the two treatment groups and of both genders (*n* = 3/group/gender). Acquisition settings and magnification were the same for each picture. Original magnification: × 400. Size bar = 20 μm. (**B**) Quantification of the number of Cc3-positive cells in tissues stained as shown in A (*n* = 3/treatment group/sex) was performed as indicated in the Materials and Methods. Results are presented as the mean percentage of Cc3 positive cells ± SD. ***, *p* < 0.0001.

The activation of Sstr3 by somatostatin/SSAs or its overexpression has been associated with the induction of apoptosis in various normal and tumor cells [13,25,26]. Given the high expression of *Sstr3* in female rats, and the volume reduction of some tumors in females treated with pasireotide, we conducted immunofluorescence with an anti-Cleaved Caspase 3 (Cc3) antibody as a marker of apoptosis. Specifically, rat tumor tissues from males and females treated with pasireotide were stained for Cc3 and the number of positive cells in tumor areas counted. We observed that while placebo-treated control rats of both sexes show virtually no apoptosis (Figure 7A), tumors of pasireotide-treated rats showed an increase in Cc3-positive cells only in female rat tumors, but not in male PTs (males pasireotide-treated vs. females pasireotide-treated, *p* < 0.0001, two-way Anova) (Figure 7B).

### 2.4. SSTR3 Expression in Human NFPTs

**Figure 8:**
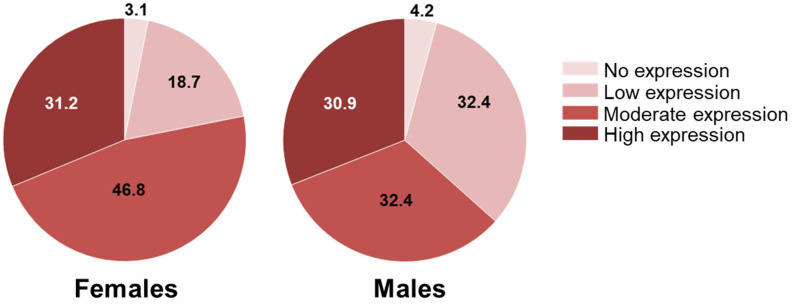
SSTR3 expression in human NFPTs. A cohort of 107 NFPT patients was previously characterized for the expression of SSTRs, including SSTR3. Utilizing the published semi-quantitative expression scoring method, tumors were divided into four categories of protein expression, as indicated. The distribution of the tumors having these scores between males and females is indicated in the percentage of the total cases.

In this study, we observed that rat NFPTs show a sex-related difference in the expression of *Sstr3*. In a previous study, we investigated the expression of SSTRs in a large cohort of human NFPTs and found SSTR3 to be the most highly expressed receptor subtype [15]. In that study, we did not stratify patients for sex. So, we went back to these data and graphed SSTR3 expression in male and female patients. The results showed that 78% of the tumors in females had moderate to high expression of SSTR3, whereas these levels of expression were seen only in 63.3% of male patients (Figure 8), demonstrating a trend for higher expression in the females, which was however not significant (*p* = 0.097).

## 3. Discussion

We here report that the multi-receptor ligand pasireotide is more effective than octreotide at inhibiting tumor growth in a representative model of endogenous NFPTs in vivo. Tumor volume was longitudinally monitored during treatment, and molecular endpoints were assessed to elucidate the mechanisms of drug response.

While tumors in placebo-treated rats increased in volume all throughout the treatment duration, those in rats treated with octreotide LAR (sandostatin^®^, Novartis Pharma GmbH, Nürnberg, Germany) or pasireotide LAR (signifor^®^, Novartis Pharma GmbH, Nürnberg, Germany) grew significantly less and in a few cases, we observed a reduction in tumor volume. The number of rats that responded to pasireotide (i.e., increase in tumor volume below 20% over the 2-month period) was six out of nine males and eight out of eight females, which was significantly higher than the number of octreotide-responsive tumors: one out of four for the males, and three out of five for the females. Accordingly, tumor shrinkage at the end of the treatment (vs. the pre-treatment volume) occurred in four out of nine males and in six out of eight females injected with pasireotide, but only in 1/5 female rats (no males showed shrinkage) upon octreotide administration. This indicates that a pan-receptor ligand is more potent than an Sstr2-preferring SSA against MENX-associated PTs.

The radiological response of the treated rats correlated with the proliferation rate of the tumors: there was a more drastic reduction in Ki67-positive cells in NFPTs of animals treated with pasireotide vs. controls than in those treated with octreotide vs. controls, further supporting the stronger anti-tumor effect of the former.

Tumor growth is the main clinical problem associated with NFPTs. Indeed, when they grow to become macroadenomas they cause symptoms due to compression of nearby structures and cannot be completely resected thereby leading to high relapse rates. Therefore, a treatment option that potently suppresses cell proliferation might be beneficial for aggressive/recurrent NFPTs, currently an orphan of effective therapy.

In vitro, pasireotide and octreotide have already been compared for their efficacy against primary human somatotropinoma (GH-secreting-PTs) [27], corticotropinoma (ACTH-secreting) [28] and also NFPT cells [17]. To elucidate the molecular mechanisms associated to PT cell response, readouts such as suppression of cell viability and hormone secretion (for the functioning tumors) were assessed in these primary cultures, and correlated with the expression of *SSTR* genes. In somatotropinomas, a higher expression of the *SSTR2* gene was associated with higher efficacy of octreotide compared with pasireotide, whereas tumors with a higher level of *SSTR5* tended to respond better to pasireotide [17]. Octreotide was less potent than pasireotide at inhibiting ACTH secretion in corticotropinomas [28], whereas in primary NFPT cells neither drug affected cell viability [17], or pasireotide was less effective than octreotide at reducing it [17]. The expression pattern of the *SSTR* genes was not always able to explain the sensitivity of human primary PT cells to SSAs, thereby suggesting that other mechanisms might be at play [17].

Primary cultures of human tumors are a useful in vitro model as they allow the assessment of the response of tumor cells to drugs in vitro, which can then be correlated to the molecular makeup of these cells, or to the clinical parameters of the patients. However, in vitro 2D cultures do not recapitulate the interaction between tumor cells and the tumor microenvironment, nor they permit the evaluation of extrinsic (in this case extra-pituitary) effects that are relevant to determine the response therapy. Our results of an anti-proliferative effect of both SSAs, and especially of pasireotide, against NFPTs in vivo, which is more potent than what has so far been reported for these tumors grown as primary cultures, highlight the limited value of in vitro drug testing studies in predicting the efficacy of anti-tumor agents at the organism level.

The antiproliferative effects of SSAs result from the induction of either growth arrest or apoptosis. It has been shown that apoptosis is mainly mediated by activation or ectopic expression of SSTR3 (among the various receptors) in several normal and tumor cell types [13,25,26]. Given that female rats express a higher basal level of *Sstr3* and some of their tumors showed shrinkage during treatment with pasireotide, we checked whether this tumor reduction was due to the induction of apoptosis. Staining with an anti-Cc3 to assess apoptosis, the antibody showed that indeed pasireotide promoted apoptosis only in female rat tumors. Therefore, pasireotide treatment was particularly effective against female rat NFPTs likely because it combined the anti-proliferative and pro-apoptotic effects associated with Sstr3 activation.

Pasireotide shows the highest affinity for SSTR5 [14], the receptor most prevalent in corticotroph tumors, and, therefore, this drug is used for the treatment of Cushing’s Disease in patients who relapse or are unsuitable for surgery [29]. In the rat tumors, while the expression of *Sstr5* at the mRNA level is high, the results at the protein level were inconclusive due to the poor performance of the antibody on rat tissues. Thus, the efficacy of pasireotide in our model is not dependent on Sstr5 expression. Similarly, the expression of SSTR5 at gene and protein levels is negligible in human primary NFPT cells [17] and primary tumor tissues [15], respectively. The shared SSTR5 expression pattern between the two species makes MENX rats a model recapitulating the cognate human tumors also in an aspect highly relevant in view of a possible clinical implementation of pasireotide for the treatment of NFPT patients.

In agreement with previously reported data on human PT tissues [22,23,24] and on primary cultures derived from these tumors [16,17], treatment with octreotide or pasireotide modulated the expression level of the receptors at mRNA and/or protein levels. Octreotide treatment of rat NFPTs led to a decrease of Sstr2 at the protein level in both sexes. A reduced level of SSTR2 has been previously observed in PT patients who underwent presurgical therapy with SSAs [22,24]. In contrast, octreotide had no effect on the mRNA level of *Sstr2*, thereby indicating that in rat PTs there is no correlation between transcript and protein product. This has also been reported for human nonpituitary NETs [30]. Octreotide also increased the expression of Sstr3 both at mRNA and protein levels, and decreased the levels of the *Sstr5* gene in rats of both sexes. Pasireotide administration increased the expression of Sstr1 at both mRNA and protein levels in both sexes, whereas it upregulated Sstr3 transcript/protein in affected males. Whether the changes in gene/protein expression upon drug treatment affect the response to therapy is difficult to assess based on the lack of biochemical treatment readouts such as the inhibition of hormone secretion.

During our studies, we observed that female rats responded better than males to both SSAs in general, and particularly well to pasireotide. The fact that PTs in female rats grow more slowly than in males, as demonstrated by both MRI and Ki67 staining, could be related to the sex-dependent sensitivity to SSAs. Additionally, the fact that tumors in female rats at baseline express significantly higher levels of *Sstr3* than those in males may account for the potent suppressive effect of pasireotide on tumor growth in female rats. Indeed, although treatment with both SSAs increased *Sstr3* gene expression in male NFPTs, their final levels were still lower than those in the control female tumors. We then investigated whether there might be a differential expression of SSTR3 based on sex also in human NFPT patients. To this aim, we analyzed a cohort of 107 NFPT patients that we previously characterized for SSTRs expression [15]. Similar to the situation in MENX rats, also female patients have a trend for a higher expression of SSTR3 than males (although not statistically significant). In human cancer patients, a sex-dependent expression of SSTR genes/proteins has not been reported to date. If confirmed in additional patients, this observation could have important clinical implications. Specifically, a sex-associated SSTR3 expression might be used as criteria to stratify patients for treatment with pasireotide and improve therapy response, given that the affinity of this drug for SSTR3 is much higher than that of first-generation SSAs. If a different SSTR3 expression based on sex holds true also for other PT cell types, these results could have broader clinical relevance given that pasireotide is currently recommended for the treatment of patients with Cushing’s disease in whom surgery was unsuccessful, and of acromegalic patients who remain uncontrolled after surgery or in whom tumor resection is not possible.

## 4. Materials and Methods

### 4.1. Rats and Treatment

Rats were maintained in agreement with general husbandry rules approved by the Helmholtz Zentrum München and all experimental procedures were conducted in accordance with committed guidelines as approved by the local government (GV-Solas; Felasa; TierschG). In vivo studies were approved by the government of Upper Bavaria, Germany (rat studies: Az. 55.2.1.54-2532-39-13 and 55.2-2532.Vet_02-18-102).

MENX rats were injected s.c. with octreotide LAR (sandostatin^®^) at doses of 20 mg/Kg body weight (bw), with pasireotide LAR (signifor^®^) at the dose of 20 mg/Kg bw, or placebo (PBS) (control group) every 28 days for a total of 28 or 56 days (see Figure 1A). Rats were treated when they were 6.5 months (28 d time course) or 5.5 months (56 d time course). Treatment protocols and optimal drug concentrations were provided by Novartis Pharma GmbH, Nürnberg, Germany, and are based on in-house data on drug administration.

### 4.2. Human Tissue Samples

Human NFPTs analyzed in this study were previously described [15]. Prior studies utilizing these specimens were approved by local ethics committees (Univ. Tübingen:456/2009BO2; Univ. Munich, TUM: 169/17S) requiring informed written patient consent.

### 4.3. Magnetic Resonance Imaging (MRI)

Tumor monitoring in rats was conducted using MRI pre-treatment (day 0) and every 14 days post-treatment (days 14, 28, 42, 56). MRI was performed on a 7 T preclinical scanner (Bruker BioSpin MRI GmbH, Ettlingen, Germany) using a ^13^C/^1^H volume resonator and a 4 channel ^1^H rat head surface coil array (both: RAPID Biomedical, Rimpar, Germany). Anesthetized animals (2.5% isoflurane, administered in pure oxygen) were placed on an animal bed in a prone position (tail ahead) and the particular surface coil was positioned over the head/tumor. For tumor volume determination, T_2_ weighted imaging was applied (sequence parameters (T2_TurboRARE): TE: 40 ms, TR: 3000 ms, averages: 2, rare factor: 8, slices: 20, slice orientation: sagittal, read orientation: rostral-caudal, slice thickness: 0.800 mm, image size (matrix): 256 × 256, field of view (FoV): 40 × 35 mm², resolution: 0.156 × 0.137 mm², fat suppression: on). The actual volume determination was performed in Osirix/Horos (Pixmeo SARL, Geneva, Switzerland; the Horos Project, Annapolis, MD, USA) to quantify and evaluate tumor growth. Regions of interest (ROIs) were manually segmented around the PTs in every slice where they appeared in the T_2_ weighted datasets; tumor volumes were finally calculated from the data (area of particular ROIs and slice thickness) using an implemented algorithm in Osirix/Horos.

### 4.4. RNA Extraction and Quantitative RT-PCR

RNA was extracted using RNeasy Mini Kit (Qiagen, Hilden, Germany) following the manufacturer’s instructions. Total RNA concentration and purity were assessed using the Nanodrop 2000 spectrophotometer (Thermo Scientific, Langenselbold, Germany). Total RNA was retro-transcribed using random hexamer primers and the cDNA First-Strand Synthesis kit (Thermo Scientific). Details regarding the quantitative PCR (qPCR) procedure used to determine the absolute expression levels of the different somatostatin receptor genes (*SSTR1–5*) have previously been reported [31]. Specific sets of primers for these genes have previously been validated and reported [31]. To control for variations in the amount of RNA used in the retro-transcription (RT) reaction and in the efficiency of the RT reaction, mRNA copy numbers of the different transcripts analyzed were adjusted by beta-actin (*ACTB*) expression (used as housekeeping gene) [31].

### 4.5. Immunohistochemistry (IHC)

From the paraffin blocks, 4-µm sections were prepared and floated onto positively charged slides. Immunostaining for SSTRs was performed by an indirect peroxidase labelling method. Briefly, sections were dewaxed, microwaved in 10 mM citric acid (pH 6.0) for 16 min at 600 W, and incubated with the anti-SSTR antibodies overnight at 4 °C (SSTR1: E4317, anti-human/rat/mouse, rabbit polyclonal, affinity-purified, concentration 1 µg/mL; SSTR2: UMB-1, anti-human/rat/mouse, rabbit monoclonal, cell culture supernatant, dilution: 1:10; SSTR3: 1308, anti-rat, rabbit polyclonal, affinity-purified, concentration 1 µg/mL; SSTR5: 6003, anti-rat, rabbit polyclonal, dilution: 1:2000 [32,33,34,35]. The anti-SSTR5 antibody did not give reliable signal on rat tissues and was therefore not included in the analysis. Detection of the primary antibody was performed using biotinylated anti-rabbit IgG followed by incubation with peroxidase-conjugated avidin (Vector ABC “Elite” kit; Vector Laboratories, Burlingame, CA, USA). Binding of the primary antibody was visualised using 3-amino-9-ethylcarbazole in an acetate buffer (BioGenex, San Ramon, CA, USA). Sections were rinsed, counterstained with Mayer’s haematoxylin, and mounted in Vectamount™ mounting medium (Vector Laboratories, Burlingame, CA, USA). Sections from rat pancreases or pituitaries served as positive controls. As negative controls, the primary antibodies were either omitted or adsorbed for 2 h at room temperature with 10 µg/mL of the peptide used for immunizations. In all cases, a complete abolition of immunostaining was observed.

Immunohistochemistry for Ki-67 (BD Pharmingen, Franklin Lakes, NJ, USA, 556003, dilution: 1:1000) and Cleaved caspase 3 (Cc3, Cell Signaling Technology, Danvers, MA, USA, 9664, dilution: 1:150) was performed using a Bond RXm system (Leica, Wetzlar, Germany, all reagents from Leica). Briefly, slides were deparaffinized using deparaffinization solution, and pretreated with Epitope retrieval solution 1 (corresponding to citrate buffer pH 6) for 20 min. Tissues were incubated with the primary antibody for 15 min at room temperature. Antibody binding was detected with a polymer refine detection kit used without post-primary reagent for the Cc3 antibody and visualized with DAB as a dark brown precipitate. Counterstaining was performed with hematoxylin.

Ki67-positive tumor cells per 100,000 µm^2^ were counted under the microscope in 3 independent areas for each tumor sample.

### 4.6. Evaluation of Immunostains for SSTRs

Immunostains for SSTRs were evaluated semi-quantitatively on acquired images. An immunoreactive score (IRS) was recorded for each section. The IRS was generated noting the intensity of the staining (no staining, 0; mild, 1; moderate, 2; and strong, 3) and the percentage of cells in the tumor area showing expression (no positive cells, 0; <10% of positive cells, 1; 10–50% of positive cells, 2; 51–80% of positive cells, 3; and >80% of positive cells, 4). The overall IRS was calculated as (percentage of positive cells) × (intensity of staining). Staining was classified as having low positivity for IRS < 3, intermediate positive for IRS 4–8, and strongly positive for IRS > 8. The slides were scored semi-quantitatively by two experienced animal pathologists (H.-Y.Y. and K.S.). Scoring was performed independently, by a double-blind method, according to the criteria reported above with an inter-observer variability ranging from 1% to 3.7%. Discrepancies were discussed among the pathologists.

### 4.7. Immunofluorescence (IF) and Quantification

Immunofluorescence analyses were performed using the formalin-fixed paraffin-embedded 4-μm sections that were deparaffinized, fixed and boiled in 10 mM citric acid (pH 6.0) for 16 min at 600 W. Afterwards, cells were permeabilized and blocked with 0.1% Triton X-100, 4% NGS and 3% BSA solution and incubated with anti-Cc3 antibody (9664s, Cell Signaling Technology, Danvers, MA, USA, dilution: 1:1000) overnight at 4 °C and alexa 555 (A-21429, Thermo Fisher Scientific, Waltham, MA, USA, dilution 1:1000) for 1h. Between each step, tissues were washed three times with TBS-T, pH 8. Lastly, tissues were stained with Hoechst 33258 (dilution 1:2000) for nuclei staining and mounted using ProLong Glass Antifade Mountant (P36984, Thermo Fisher Scientific, Waltham, MA USA). Images were obtained using a laser scanning confocal microscope (Olympus FluoView 1200; Olympus Corporation) equipped with an Olympus UPlanSAPO × 60 1.35 and an UPlanSAPO ×40 1.25 solid immersion lens oil immersion objective (Olympus). Cc3-positive cells were counted in 3 independent and representative high Power Fields (HPF) for each tumor sample under the confocal microscope (*n* = 2/treatment group/sex).

### 4.8. Statistical Analysis

Linear mixed-effects (LME) models were applied for longitudinal analysis of tumor volume growth with absolute tumor volume at day 0 used for scaling subsequent measurements of each individual. Relative volumes were transformed by natural logarithms for use as model outcomes in order to meet the normal distributional assumptions. Linear and quadratic growth predictors and interactions were considered for significance testing, performed by the F-test, with results presented as the mean ± standard error of the mean (SEM). Statistical significance between two series of data (e.g., Figure 1, Figure 2 and Figure 3) was determined by one-way ANOVA. A *p*-value < 0.05 was considered statistically significant.

## 5. Conclusions

NFPTs are the most common PT subtype, are often invasive and not amenable to complete surgical resection. This leads to high relapse rates. As no effective medical therapy for NFPTs currently exists, the identification of novel treatments is of high clinical relevance. Our in vivo therapy studies in the only spontaneous model of NFPTs (MENX rats) demonstrate that an SSA that preferentially binds to multiple SSTRs has superior antitumor potential in the treatment of NFPTs compared to an SSTR2-directed analog. It is noteworthy that therapy-response in this model is associated with *Sstr3* expression. These results provide the rationale for the clinical investigation of pasireotide against NFPT patients with residual and recurrent disease, as well as with other NET types having high SSTR3 expression. Furthermore, we unveil a possible sex-dependent SSTR3 expression, which might be predictive of drug efficacy and deserves to be further explored not only in PTs but also in other NETs.

## Data Availability

Not applicable.

## Supplementary Materials

The following are available online at https://www.mdpi.com/article/10.3390/cancers13123097/s1, Figure S1. MRI of the pituitary glands of male rats representative of the three treatment groups. Figure S2. Trace plots of relative tumor volume in the various group treated for 56d. Figure S3. Mean relative tumor volume of the various treatments. Figure S4. Population analysis of experimental tumor growth kinetics. Figure S5. Tumor cell proliferation at the end of the treatment. Figure S6. Relationship among qRT-PCR data, Ki67 proliferation rate and tumor volume in the control group. Table S1. List of the rats used for the study. Table S2. Longitudinal data of the rats treated with placebo (control). Table S3. Longitudinal data of rats treated with octreotide LAR. Table S4. Longitudinal data of rats treated with pasireotide LAR.
