# Supplementary material for: Gender-Specific Efficacy Revealed by Head-to-Head Comparison of Pasireotide and Octreotide in a Representative In Vivo Model of Nonfunctioning Pituitary Tumors
Source: Cancers (Basel). 2021 Jun 21;13(12):3097. doi: 10.3390/cancers13123097 (PMC8235746; doi:10.3390/cancers13123097)
Supplement: Supplementary file 1 [file cancers-13-03097-s001.zip › cancers-1245150-supplementary.pdf]

cancers

Supplementary

Gender-Specific Efficacy Revealed by Head-to-Head Comparison of Pasireotide and Octreotide in a Representative In Vivo Model of Nonfunctioning Pituitary Tumors

Sebastian Gulde et. al

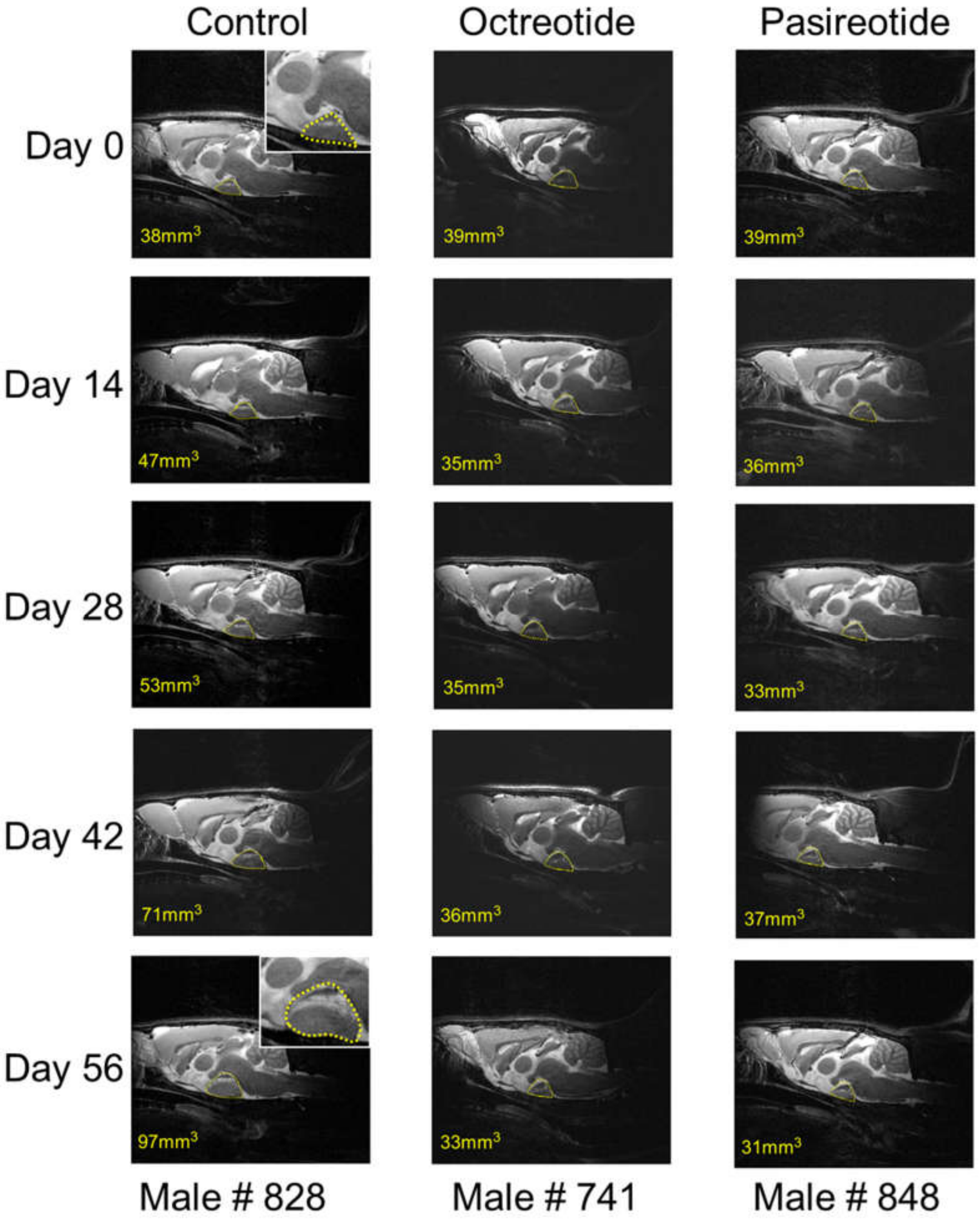

Figure S1. MRI of the pituitary glands of male rats representative of the three treatment groups. Screenshots of the longitudinal MRI scans of three male rats were taken always through the largest gland diameter. Tumor volume at the different time points is indicated. The dotted line illustrates the pituitary gland.

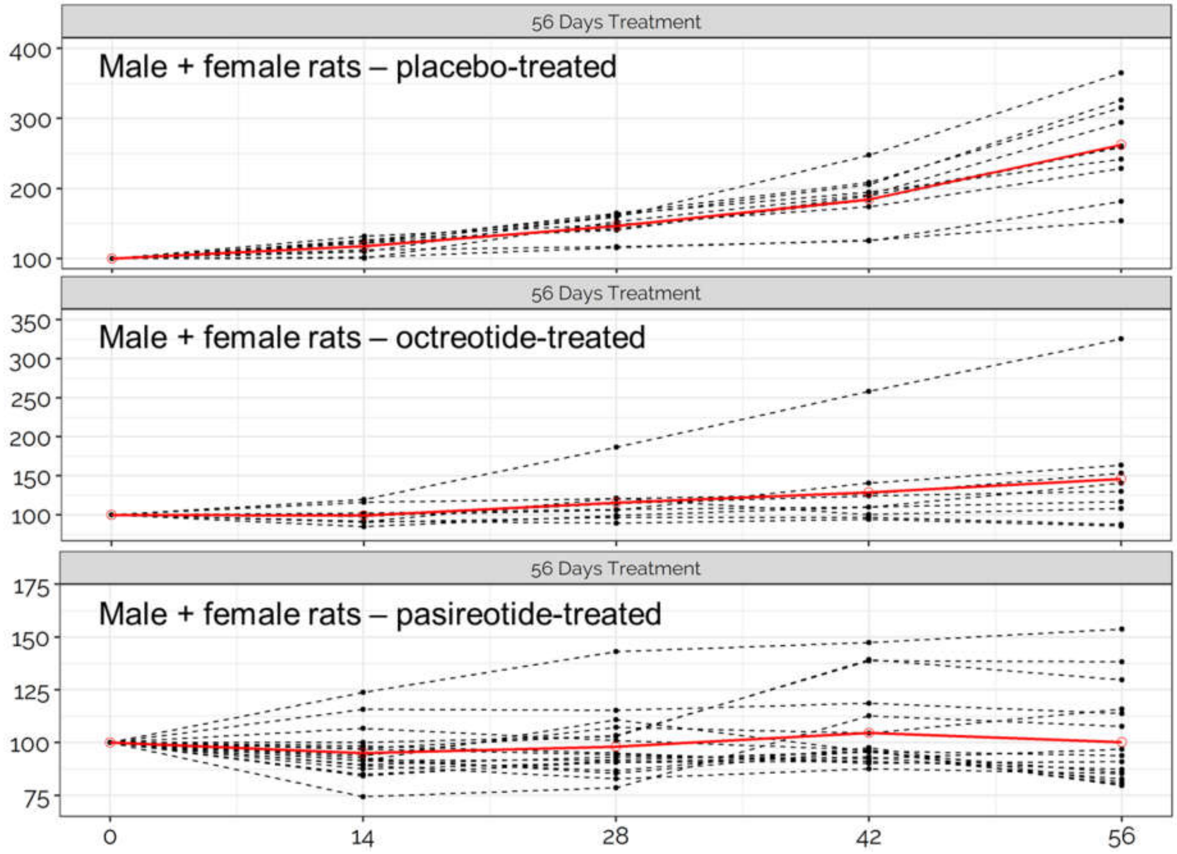

Figure S2. Trace plots of relative tumor volume in the various group treated fro 56d. Trace plots of tumor volume for mutant male and female rats together, relative to volume at day 0 for each animal. Details about individual rats and the statistical analyses are reported in Figure 2.

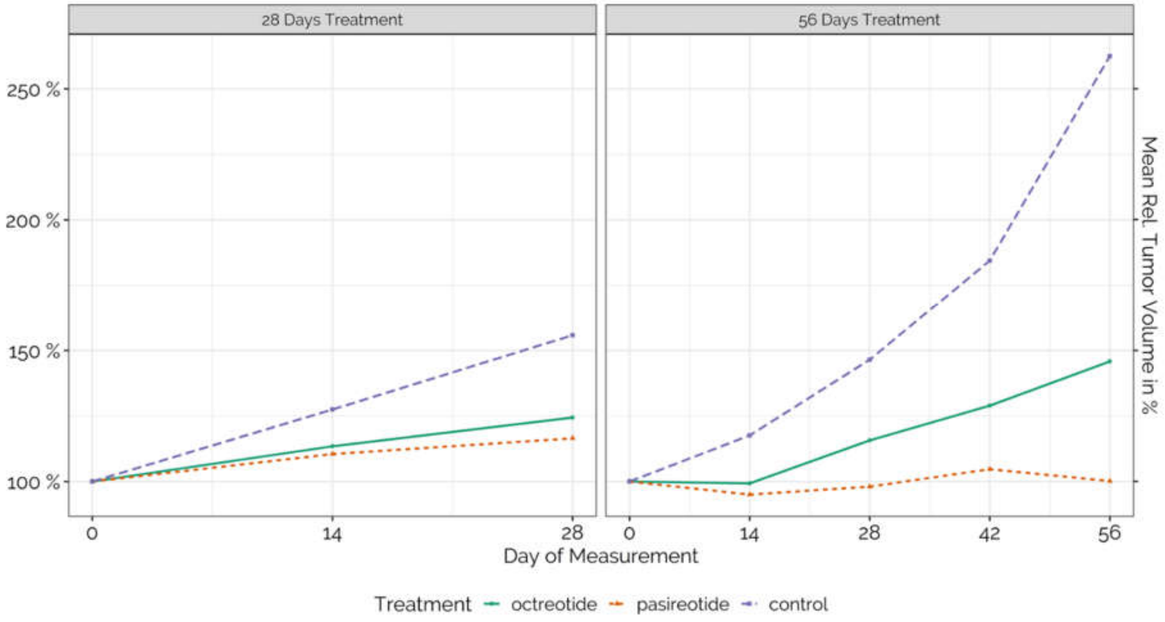

Figure S3. Mean relative tumor volume changes of the various treatment groups. Mean value slope of relative tumor volume for 7.5-month-old mutant rats belonging to the different treatment groups. Left side: 28d treated rats. right side: 56d treated rats

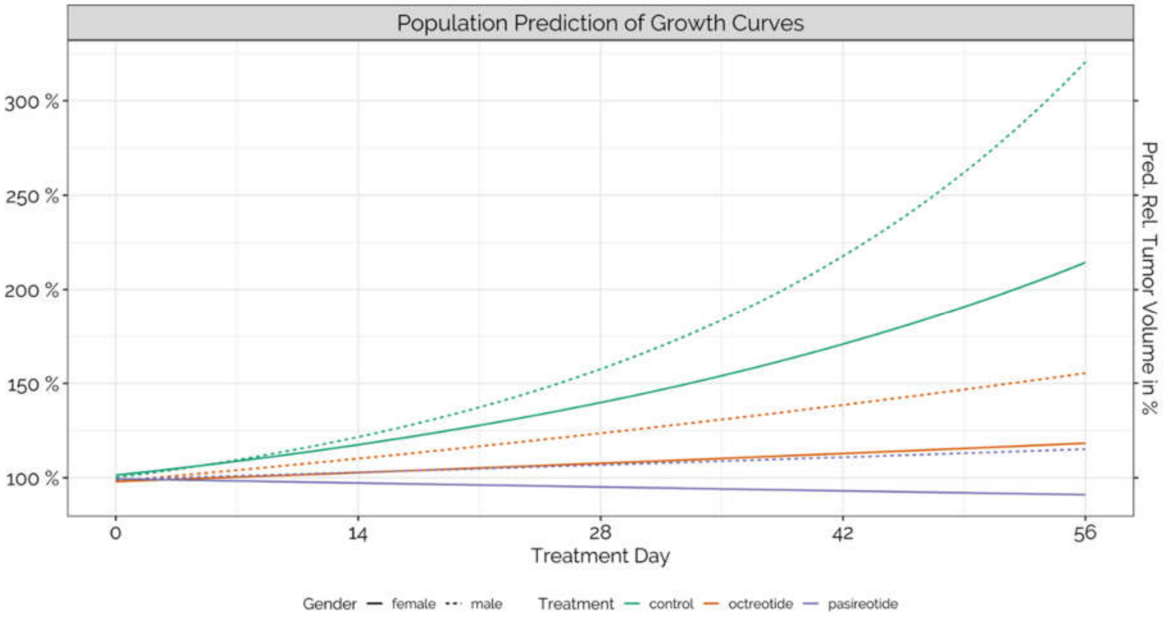

.

Figure S4. Population analysis of experimental tumor growth kinetics. For control rats, a LME model with mean structure of log(rel_volume) ~ 1 + day + male + male × day + day^2 + male × day^2 and random effect structure ~ -1 + day achieved the best fit. In contrast, octreotide and pasireotide both had a mean structure of log(rel_volume) ~ 1 + day + male + male × day with random effects structure of -1 + day, -1 + day + day^2, respectively. All parameters are estimated by restricted maximum likelihood estimation. The fitted average rel. tumor volume profiles for different gender and treatment are shown.

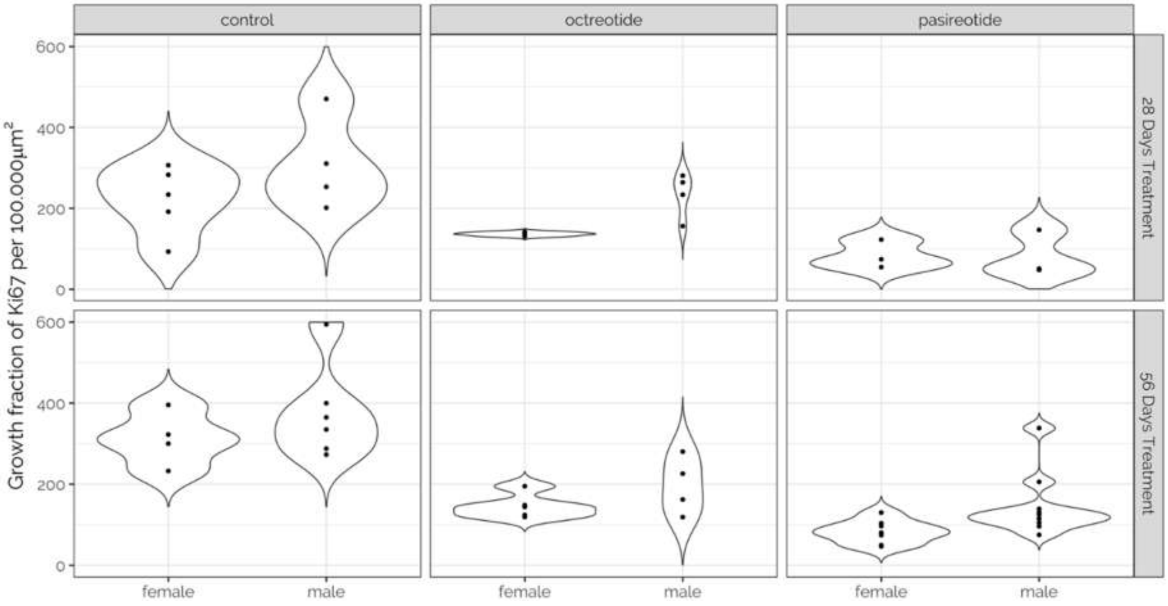

Figure S5. Tumor cell proliferation at the end of the treatment. The number of Ki67-positive cells/100.000µm^2 was counted in a subset of rat PTs (*n* = 3 to 8) of both genders and for each group at the end of treatment. Shown are the numbers for 28d treated rats (upper row), and for 56d treated animals (bottom row). The distribution is shown as a violin plot.

A

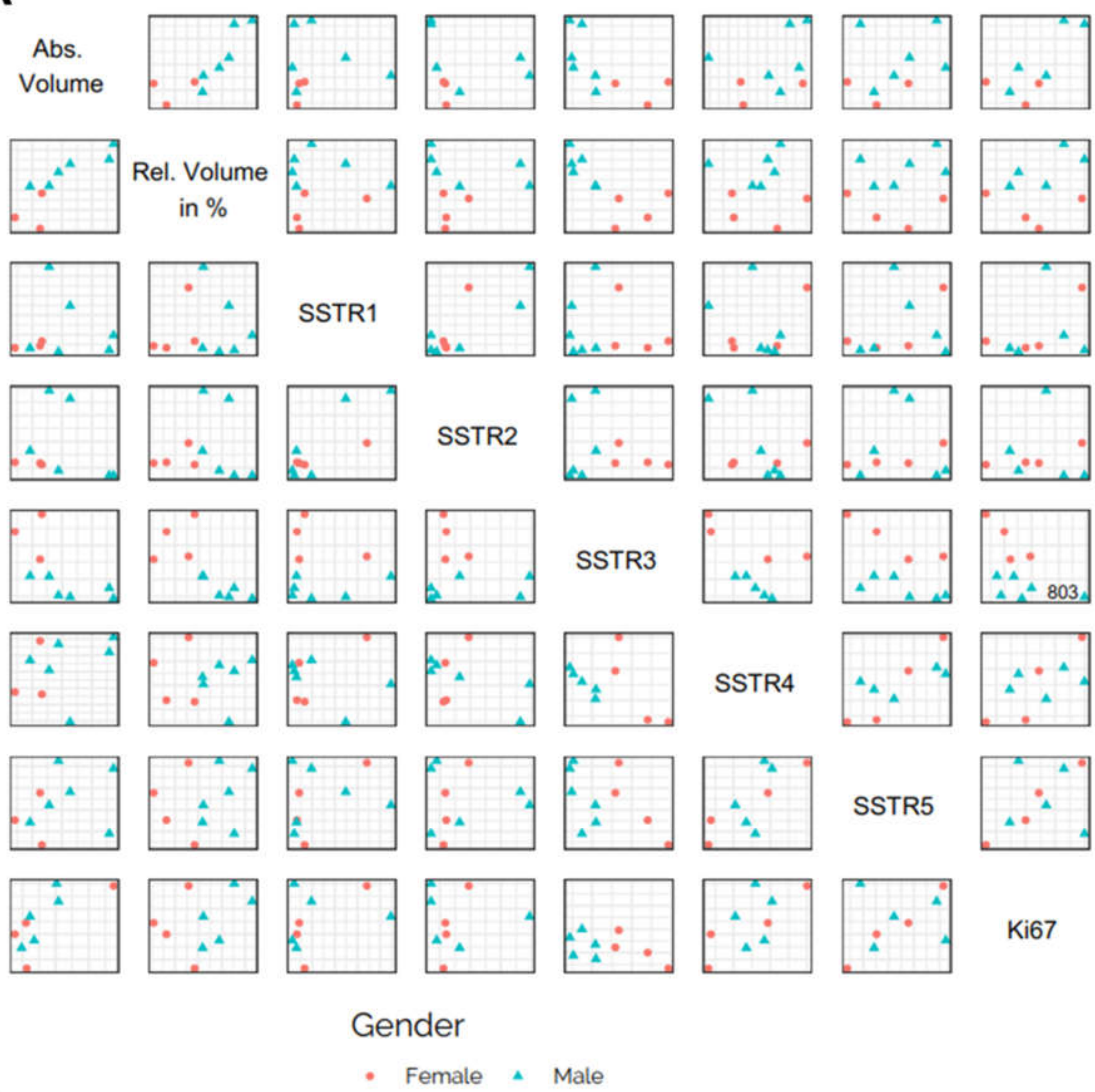

B

Response	Covariate	Estimate	Outlier	p-value	Significance
Abs. Volume	Rel. Volume	$5.597*10^{-4}$	802	0.0044	**
Abs. Volume	SSTR3	$-6.675*10^{-6}$	802	0.055	.
Abs. Volume	Ki67	$6.771*10^{-4}$	802,803	0.033	*
Rel. Volume	SSTR3	-0.011	—	0.019	*
Rel. Volume	Gender	101.98	—	0.0048	*

Figure S6. Relationship among qRT-PCR data, Ki67 proliferation rate and tumor volume in the control group. (**A**) Scatter matrix of Sstr gene expression by qRT-PCR, Ki67 staining and absolute or relative tumor volume in rats of the control group. (**B**) Table summarizing the relationship of some parameters shown in A. Red arrows point to the significant correlations mentioned in the Results.

Supplementary Table 1: List of the rats used for the study

Rat Nr.:	Treatment (days)	Gender	Age
**673**	Placebo control (28d)	M	7.5
**674**	Placebo control (28d)	M	7.5
**758**	Placebo control (28d)	M	8.5
**760**	Placebo control (28d)	M	8.5
**803**	Placebo control (2x28d)	M	8
**823**	Placebo control (2x28d)	M	7.5
**812**	Placebo control (2x28d)	M	8
**813**	Placebo control (2x28d)	M	8
**828**	Placebo control (2x28d)	M	7.5
**829**	Placebo control (2x28d)	M	7.5
**679**	Placebo control (28d)	F	7.5
**681**	Placebo control (28d)	F	7.5
**683**	Placebo control (28d)	F	7.5
**772**	Placebo control (28d)	F	8.5
**773**	Placebo control (28d)	F	8.5
**802**	Placebo control (2x28d)	F	8
**818**	Placebo control (2x28d)	F	7.5
**807**	Placebo control (2x28d)	F	8
**808**	Placebo control (2x28d)	F	8
**696**	Sandostatin (28d)	M	7.5
**707**	Sandostatin (28d)	M	7.5
**698**	Sandostatin (28d)	M	7.5
**708**	Sandostatin (28d)	M	7.5
**731**	Sandostatin (2x28d)	M	7.5
**740**	Sandostatin (2x28d)	M	7.5
**741**	Sandostatin (2x28d)	M	7.5
**887**	Sandostatin (2x28d)	M	7.5
**703**	Sandostatin (28d)	F	7.5
**704**	Sandostatin (28d)	F	7.5
**706**	Sandostatin (28d)	F	7.5
**720**	Sandostatin (28d)	F	7.5
**736**	Sandostatin (2x28d)	F	7.5
**737**	Sandostatin (2x28d)	F	7.5
**747**	Sandostatin (2x28d)	F	7.5
**891**	Sandostatin (2x28d)	F	7.5
**897**	Sandostatin (2x28d)	F	7.5
**665**	Signifor (28d)	M	7.5
**686**	Signifor (28d)	M	7.5
**675**	Signifor (28d)	M	7.5
**729**	Signifor (2x28d)	M	7
**730**	Signifor (2x28d)	M	7.5
**743**	Signifor (2x28d)	M	7.5
**837**	Signifor (2x28d)	M	8
**847**	Signifor (2x28d)	M	7.5
**848**	Signifor (2x28d)	M	7.5
**849**	Signifor (2x28d)	M	7.5
**850**	Signifor (2x28d)	M	7.5
**851**	Signifor (2x28d)	M	7.5
**852**	Signifor (2x28d)	M	7.5
**680**	Signifor (28d)	F	7.5
**684**	Signifor (28d)	F	7.5
**678**	Signifor (28d)	F	7.5
**732**	Signifor (2x28d)	F	7.5
**735**	Signifor (2x28d)	F	7.5
**739**	Signifor (2x28d)	F	7.5
**771**	Signifor (2x28d)	F	7.5
**831**	Signifor (2x28d)	F	8
**832**	Signifor (2x28d)	F	8
**835**	Signifor (2x28d)	F	8
**842**	Signifor (2x28d)	F	7.5

Supplemental Table 2. Longitudinal data of rats treated with placebo (control)

Rat	Gender	Day	Abs. Volume	Rel. Volume	SUV
673	M	0	0.068	100.00	0.833
673	M	14	0.087	129.25	0.708
673	M	28	0.122	179.91	0.065
674	M	0	0.038	100.00	0.943
674	M	14	0.048	126.79	0.589
674	M	28	0.064	169.50	0.067
679	F	0	0.112	100.00	1.024
679	F	14	0.122	108.64	1.304
679	F	28	0.127	113.36	0.079
681	F	0	0.08	100.00	0.634
681	F	14	0.097	120.62	0.58
681	F	28	0.107	132.42	0.051
683	F	0	0.095	100.00	0.722
683	F	14	0.102	107.44	1.304
683	F	28	0.12	126.10	0.05
758	M	0	0.106	100.00	2.12
758	M	14	0.136	127.54	NA
758	M	28	0.218	204.51	2.434
760	M	0	0.049	100.00	1.718
760	M	14	0.056	114.90	NA
760	M	28	0.062	126.33	2.07
772	F	0	0.067	100.00	0.837
772	F	14	0.104	155.74	NA
772	F	28	0.108	161.25	0.793
773	F	0	0.067	100.00	0.838
773	F	14	0.105	156.53	NA
773	F	28	0.127	188.72	0.945
802	F	0	0.164	100.00	2.971
802	F	14	0.216	131.73	NA
802	F	28	0.244	148.54	1.587
802	F	42	0.285	173.69	NA
802	F	56	0.375	228.62	4.051
803	M	0	0.048	100.00	2.539
803	M	14	0.059	124.48	NA
803	M	28	0.079	164.64	2.157
803	M	42	0.1	208.58	NA
803	M	56	0.151	315.06	1.779
807	F	0	0.042	100.00	1.076
807	F	14	0.048	112.29	NA
807	F	28	0.049	116.79	0.873
807	F	42	0.053	125.06	NA
807	F	56	0.077	181.32	1.076
808	F	0	0.072	100.00	1.003
808	F	14	0.073	101.53	NA
808	F	28	0.083	115.18	0.874
808	F	42	0.09	125.63	NA
808	F	56	0.11	153.76	1.24
812	M	0	0.046	100.00	1.265
812	M	14	0.046	100.44	NA
812	M	28	0.07	152.72	0.932
812	M	42	0.088	191.29	NA
812	M	56	0.135	294.34	1.718
813	M	0	0.047	100.00	1.167
813	M	14	0.057	121.66	NA
813	M	28	0.067	142.89	1.07
813	M	42	0.086	183.44	NA
813	M	56	0.123	260.30	1.833
818	F	0	0.047	100.00	1.452
818	F	14	0.051	109.42	NA
818	F	28	0.076	163.17	1.054
818	F	42	0.091	194.86	NA
818	F	56	0.113	241.54	1.291
823	M	0	0.062	100.00	1.523
823	M	14	0.078	125.72	NA
823	M	28	0.1	161.09	1.108
823	M	42	0.128	205.15	NA
823	M	56	0.203	326.37	1.375
828	M	0	0.037	100.00	0.883
828	M	14	0.047	126.13	NA
828	M	28	0.053	140.53	0.668
828	M	42	0.071	189.07	NA
828	M	56	0.097	258.67	0.711
829	M	0	0.057	100.00	2.925
829	M	14	0.07	122.20	NA
829	M	28	0.091	159.27	1.867
829	M	42	0.142	247.73	NA
829	M	56	0.209	365.04	3.467

Supplemental Table 3. Longitudinal data of rats treated with octreotide LAR

Rat	Gender	Day	Abs. Volume	Rel. Volume	SUV
696	M	0	0.128	100.00	1.079
696	M	14	0.166	129.11	NA
696	M	28	0.236	183.66	0.815
698	M	0	0.086	100.00	1.922
698	M	14	0.123	143.51	NA
698	M	28	0.138	161.87	1.096
703	F	0	0.044	100.00	0.652
703	F	14	0.044	99.10	NA
703	F	28	0.05	112.14	0.477
704	F	0	0.066	100.00	1.33
704	F	14	0.077	117.02	NA
704	F	28	0.085	128.72	0.678
706	F	0	0.06	100.00	0.788
706	F	14	0.063	104.49	NA
706	F	28	0.062	102.49	0.564
707	M	0	0.043	100.00	1.005
707	M	14	0.041	96.26	NA
707	M	28	0.039	92.06	0.505
708	M	0	0.037	100.00	0.714
708	M	14	0.045	121.83	NA
708	M	28	0.045	120.49	0.52
720	F	0	0.073	100.00	0.858
720	F	14	0.07	95.91	NA
720	F	28	0.069	93.86	0.624
731	M	0	0.093	100.00	1.067
731	M	14	0.112	119.70	NA
731	M	28	0.174	186.51	0.684
731	M	42	0.241	257.92	NA
731	M	56	0.304	325.59	0.863
736	F	0	0.102	100.00	0.759
736	F	14	0.092	90.57	NA
736	F	28	0.098	96.56	0.622
736	F	42	0.099	96.86	NA
736	F	56	0.089	87.72	0.564
737	F	0	0.101	100.00	0.707
737	F	14	0.092	91.39	NA
737	F	28	0.122	121.19	0.64
737	F	42	0.102	100.59	NA
737	F	56	0.109	108.22	0.472
740	M	0	0.043	100.00	0.725
740	M	14	0.044	102.10	NA
740	M	28	0.046	106.99	0.601
740	M	42	0.047	109.79	NA
740	M	56	0.06	140.79	0.566
741	M	0	0.039	100.00	1.409
741	M	14	0.035	91.71	NA
741	M	28	0.034	89.38	0.679
741	M	42	0.036	94.04	NA
741	M	56	0.033	85.75	0.611
747	F	0	0.052	100.00	0.597
747	F	14	0.044	84.59	NA
747	F	28	0.051	99.23	0.395
747	F	42	0.057	109.63	NA
747	F	56	0.061	116.96	0.513
887	M	0	0.044	100.00	1.625
887	M	14	0.043	99.31	NA
887	M	28	0.046	106.18	0.628
887	M	42	0.061	140.50	NA
887	M	56	0.071	163.62	1.05
891	F	0	0.052	100.00	0.968
891	F	14	0.061	116.19	NA
891	F	28	0.063	120.57	0.497
891	F	42	0.066	126.67	NA
891	F	56	0.081	153.52	0.974
897	F	0	0.135	100.00	1.225
897	F	14	0.132	98.07	NA
897	F	28	0.154	114.34	0.783
897	F	42	0.167	124.15	NA
897	F	56	0.175	130.09	1.152

Supplemental Table 4. Longitudinal data of rats treated with pasireotide LAR

Rat	Gender	Day	Abs. Volume	Rel. Volume	SUV
665	M	0	0.107	100	2.128
665	M	14	0.133	124.461	1.144
665	M	28	0.174	163.261	0.121
675	M	0	0.062	100	0.727
675	M	14	0.08	130.519	0.438
675	M	28	0.087	142.045	0.053
678	F	0	0.085	100	0.638
678	F	14	0.082	95.882	0.647
678	F	28	0.093	109.882	0.051
680	F	0	0.097	100	0.604
680	F	14	0.094	97.104	0.417
680	F	28	0.083	86.143	0.048
684	F	0	0.071	100	0.565
684	F	14	0.075	105.791	0.366
684	F	28	0.065	91.243	0.056
686	M	0	0.038	100	1.354
686	M	14	0.042	109.115	0.705
686	M	28	0.041	106.25	0.074
729	M	0	0.048	100	1.551
729	M	14	0.042	87.526	NA
729	M	28	0.044	90.644	0.735
729	M	42	0.044	91.06	NA
730	M	0	0.045	100	0.124
730	M	14	0.045	100	NA
730	M	28	0.046	102.882	0.955
730	M	42	0.063	139.246	NA
730	M	56	0.058	129.712	1.068
732	F	0	0.045	100	0.783
732	F	14	0.041	91.391	NA
732	F	28	0.05	110.817	0.857
732	F	42	0.043	96.026	NA
732	F	56	0.042	93.819	0.55
735	F	0	0.046	100	0.609
735	F	14	0.039	84.934	NA
735	F	28	0.042	90.83	0.628
735	F	42	0.043	93.231	NA
735	F	56	0.038	82.751	0.54
739	F	0	0.087	100	1.104
739	F	14	0.086	98.393	NA
739	F	28	0.083	95.063	0.72
739	F	42	0.078	90.126	NA
739	F	56	0.079	90.93	0.439
743	M	0	0.047	100	0.871
743	M	14	0.035	74.364	NA
743	M	28	0.037	78.602	0.715
743	M	42	0.053	112.712	NA
743	M	56	0.051	107.627	0.718
771	F	0	0.032	100	0.601
771	F	14	0.034	106.832	NA
771	F	28	0.032	100.932	0.357
771	F	42	0.031	96.584	NA
771	F	56	0.026	79.814	0.596
831	F	0	0.057	100	1.04
831	F	14	0.048	84.211	NA
831	F	28	0.052	91.754	0.449
831	F	42	0.055	95.965	NA
831	F	56	0.045	79.649	0.917
832	F	0	0.039	100	0.899
832	F	14	0.045	115.803	NA
832	F	28	0.044	115.285	0.366
832	F	42	0.046	118.653	NA
832	F	56	0.044	113.731	0.883
835	F	0	0.087	100	1.05
835	F	14	0.081	93.887	NA
835	F	28	0.093	107.266	0.969
835	F	42	0.091	104.498	NA
835	F	56	0.101	115.917	0.958
837	M	0	0.041	100	0.809
837	M	14	0.04	97.304	NA
837	M	28	0.042	103.431	0.645
837	M	42	0.057	138.725	NA
837	M	56	0.056	138.235	1.352
842	F	0	0.058	100	0.932
842	F	14	0.053	91.522	NA
842	F	28	0.053	91.696	0.798
842	F	42	0.055	95.502	NA
842	F	56	0.05	85.813	0.868
847	M	0	0.038	100	0.785
847	M	14	0.035	91.361	NA
847	M	28	0.033	86.649	0.537
847	M	42	0.037	97.644	NA
847	M	56	0.031	81.152	0.971
848	M	0	0.038	100	0.954
848	M	14	0.036	92.727	NA
848	M	28	0.033	85.455	0.411
848	M	42	0.037	96.104	NA
848	M	56	0.031	80.26	0.796
849	M	0	0.038	100	0.56
849	M	14	0.034	89.529	NA
849	M	28	0.036	93.194	0.448
849	M	42	0.035	92.408	NA
849	M	56	0.037	96.859	0.863
850	M	0	0.044	100	0.884
850	M	14	0.039	89.245	NA
850	M	28	0.036	82.838	0.527
850	M	42	0.038	87.643	NA
850	M	56	0.037	85.355	0.837
851	M	0	0.042	100	1.335
851	M	14	0.04	96.867	NA
851	M	28	0.039	94.217	0.432
851	M	42	0.038	91.084	NA
851	M	56	0.036	87.229	0.734
852	M	0	0.053	100	0.773
852	M	14	0.066	123.783	NA
852	M	28	0.076	143.071	0.475
852	M	42	0.079	147.378	NA
852	M	56	0.082	153.745	1.172
